# Differential expression and clinical significance of angiotensin (1-7)/Ang II and Elabela/Ang II ratios in patients with acute heart failure and type I cardiorenal syndrome

**DOI:** 10.3389/fmed.2026.1912581

**Published:** 2026-09-21

**Authors:** Mengdi Zheng, Biqi Chen, Yang Zhou, Ruiyang He, Zhi Wang

**Affiliations:** 1 Department of Cardiovascular Medicine, The Affiliated Brain Hospital of Nanjing Medical University, Nanjing, China; 2 Department of Physical Diagnosis, The Affiliated Brain Hospital of Nanjing Medical University, Nanjing, China; 3 Department of Cardiovascular Medicine, The Fourth Affiliated Hospital of Nanjing Medical University, Nanjing, China

**Keywords:** acute heart failure, angiotensin (1-7), cardiorenal syndrome, elabela, renin-angiotensin-aldosterone system (RAAS)

## Abstract

**Introduction:**

This study assesses the serum levels of Elabela (ELA/Apela) and apelin receptor (APJ), angiotensin II (Ang II) and angiotensin-converting enzyme (ACE), as well as Ang (1–7) and ACE2, in patients with acute heart failure (AHF) and type I cardiorenal syndrome (CRS).

**Methods:**

A total of 31 AHF patients, 38 type I CRS patients and 31 healthy controls were enrolled. Serum concentrations of ELA, APJ, Ang II, ACE, Ang (1-7) and ACE2 were measured using ELISA. ROC curves were used to assess the diagnostic performance of the ratios of Ang (1-7)/Ang II and ELA/Ang II for AHF and type I CRS.

**Results:**

The mean ratios of Ang (1-7)/Ang II and ELA/Ang II increased in both AHF and CRS groups compared to the control group. However, the ratio of ELA/Ang II significantly decreased in the CRS group compared to the AHF group. The ratio of ELA/Ang II negatively correlated with creatinine and positively correlated with eGFR in CRS patients. The AUC for Ang (1-7)/Ang II in diagnosing AHF and type I CRS was 0.7128 and 0.6978, respectively. The AUC for ELA/Ang II in diagnosing AHF and type I CRS was 0.8783 and 0.7530, respectively.

**Conclusion:**

The ELA/Ang II ratio can be used as an additional indicator for diagnosing AHF or type I CRS. Although the ELA/Ang II ratio is particularly well correlated with eGFR in patients with type I CRS, the value of the ratio in evaluating the degree of renal impairment in these patients remains to be further studied.

## 1 Introduction

Cardiorenal syndrome (CRS) can be defined as pathophysiological disorders of the heart and kidneys, where acute or chronic dysfunction in one organ may induce acute or chronic dysfunction in the other (1, 2). CRS is classified into five subtypes, among which type I CRS, also known as acute CRS, involves rapid deterioration of cardiac function leading to acute renal dysfunction (3, 4). The renin-angiotensin-aldosterone system (RAAS) is overactivated, resulting in vasoconstriction, sodium and water retention and myocardial fibrosis, which mediates the occurrence and progression of heart failure. RAAS activation is considered the main pathogenesis of type I CRS (5). Recently, it has been demonstrated that the angiotensin-converting enzyme 2 (ACE2)-angiotensin (Ang) (1-7)-Mas receptor (MasR) axis can antagonise the harmful effects of the ACE-AngII-AT1R axis in the cardiorenal system (6). ACE2 degrades Ang II and generates Ang (1-7) (7), which binds to the G protein-coupled receptor Mas (8), exerting vasodilatory, anti-proliferative, anti-inflammatory, oxidative stress-reducing, fibrosis-inhibiting and antithrombotic effects, thereby antagonising the toxic effects of Ang II (9).

Apela, also known as Elabela (ELA), is a newly discovered novel ligand of the apelin receptor (APJ) (10, 11). Apela, apelin and APJ together constitute the apelinergic system (12). Numerous studies have shown that this system has multiple effects, including regulating body fluid balance, dilating blood vessels, enhancing myocardial contractility, reducing myocardial fibrosis and improving renal function. It is a potential target for treating heart failure and CRS (13–17). Previous research has found that ELA downregulates the expression of ACE, thereby limiting the production of Ang II, while Apelin upregulates the expression of ACE2, which hydrolyses Ang II into Ang (1-7) and thereby reduces the level of Ang II (18). This suggests an interaction between the apelinergic system and the renin-angiotensin (RAS), as well as the non-classical RAS system. Currently, there are limited studies on the serum expression differences of circulating Apela and APJ, Ang II and ACE, as well as Ang (1–7) and ACE2, in patients with AHF and type I CRS. This study assesses the serum levels of the aforementioned biomarkers in patients with AHF and type I CRS, examining the differences and clinical significance of ELA/Ang II and Ang (1–7)/Ang II ratios in these conditions.

## 2 Materials and methods

### 2.1 Subjects

This study included 31 patients with AHF and 38 patients with type I CRS, all hospitalised for the first time due to an AHF attack from 2022 to 2025, and none had taken RAAS inhibitors before hospitalisation. The diagnostic criteria for patients with AHF were based on the “2021 ESC Guidelines for the Diagnosis and Treatment of Acute and Chronic Heart Failure” (19). Patients exhibited typical symptoms of AHF, with elevated N-terminal pro-B-type natriuretic peptide (NT-proBNP) levels exceeding the diagnostic threshold of 125 pg/mL, alongside echocardiographic findings. The enrolled patients in this study included heart failure with preserved ejection fraction (EF) (HFpEF), mildly reduced EF (HFmrEF) and reduced EF (HFrEF), which were defined as EF ≥ 50%, > 40% but < 50% and ≤ 40%, respectively. Furthermore, patients with type I CRS had acute kidney injury, whose diagnostic criteria were based on those established by the Kidney Disease Improvement in Global Outcomes: an increase in serum creatinine greater than or equal to 0.3 mg/dL (≥26.4 μmol/L) within 48 h, combined with an increase of at least 50% (1.5 times higher than baseline) (20). Furthermore, 31 healthy subjects matched for age and gender were selected as a healthy control group. The exclusion criteria were as follows: patients with malignant arrhythmias, haemodynamic instability, pregnancy, severe hepatic dysfunction, concurrent tumours and autoimmune system diseases. All selected participants signed the informed consent form. The study was approved by the Ethics Committee of the Brain Hospital Affiliated to Nanjing Medical University.

### 2.2 Clinical baseline data

The subjects' current medical history, past medical history, clinical symptoms, positive physical signs, gender, age, blood pressure, heart rate, blood routine test, urine routine test, stool routine test, electrocardiogram and other clinical baseline data were recorded.

### 2.3 Biochemical tests

After the subjects were enrolled, venous blood samples were collected into vacuum tubes containing clot activator and separator gel. The tubes were incubated for 30 min at room temperature and centrifuged at 3,000×g for 15 min at 4 ℃. The upper sera were extracted for routine biochemical tests using an Olympus automatic biochemical analyser. Additionally, the serum NT-proBNP concentration was detected using an Elecsys 2010 analyser and a chemiluminescence method. To avoid the effect of medicine on endogenous RAAS, 3 mL of venous blood was taken immediately from each hospitalised patient before starting treatment. The serum was extracted and stored at −80 °C for future detection of ELA, APJ, Ang II, ACE, Ang (1–7) and ACE2. The concentrations of human serum ACE, Ang (1–7) and ACE2 were detected using a quantitative enzyme-linked immunosorbent assay (ELISA) kit purchased from Chengdu Yuannuo Tiancheng Technology Co., Limited (Cat: YNL12278-96 T, YNL11539-96 T and YMS10272-A) with a determination range of 100–16,000 pg/mL, 5–160 pg/mL and 1–120 pg/mL, respectively. The sensitivity of the ACE assay was <100 pg/mL, with intra- and inter-assay CVs of both <10%. The sensitivity of the Ang (1–7) assay was 1 pg/mL, with intra- and inter-assay CVs of both <10%. The sensitivity of the ACE2 assay was <1 pg/mL, with intra- and inter-assay CVs of both <15%. Furthermore, the ELISA kit for detecting serum ELA was purchased from Peninsula Laboratories (Cat: S-1508) with a maximum detection concentration of 100,000 pg/mL. The sensitivity of the ELA assay was 88 pg/mL, with intra- and inter-assay CVs of 13.86% and 9.51%, respectively. The ELISA kits for testing serum APJ with a detection range of 23.5–1,500 pg/mL and Ang II with a range of 1–40 pg/mL were purchased from Guangzhou Sijia Biotechnology Co., Limited (Cat: CSB-EL001910HU) and Guangzhou Kangkaixin Biotechnology Co., Limited (Cat: MM-0004H1), respectively. The sensitivity of the APJ assay was 5.8 pg/mL, with intra- and inter-assay CVs of <8% and 10%, respectively. Additionally, the sensitivity of the Ang II assay was <1 pg/mL, with intra- and inter-assay CVs of <10% and 12%, respectively. The laboratory technicians performing the ELISA assays were blinded to the participants' clinical status.

### 2.4 Echocardiography

Using a cardiac ultrasound diagnostic device, with the probe frequency set at 5 MHz, the subjects were placed in a left lateral position, and the probe was positioned beside the sternum to examine the patients' apex. The subjects were then placed in a supine position, and the probe was placed in the suprasternal notch. Long-axis views were taken to scan the left ventricle, including the size of the cardiac chambers and the tissue structure. The probe was placed at the apex to comprehensively examine various cardiac views, cardiac pulsation, blood flow and valve structure. The following parameters were measured: left ventricular end-systolic diameter (LVESD) and left ventricular end-diastolic diameter (LVEDD); left atrial diameter (LAD) and right atrial diameter (RAD); left ventricular ejection fraction (LVEF) was measured using the Simpson method.

### 2.5 Statistical analyses

Baseline data analysis was conducted using IBM SPSS Statistics 27 software. ELISA experimental results were plotted using GraphPad Prism 10 statistical software. Measurement data following a normal distribution were expressed as mean ± standard deviation, and inter-group comparisons were performed using one-way analysis of variance. When the overall difference was statistically significant (*P* < 0.05), Tukey's test was used for *post hoc* multiple comparisons. Measurement data not following a normal distribution were expressed as median and interquartile range (Q25, Q75), and inter-group comparisons were performed using the Kruskal–Wallis H test. When the overall difference was statistically significant (*P* < 0.05), Dunn's test was used for *post hoc* multiple comparisons. Enumeration data were expressed as frequency and percentage (n, %), and inter-group comparisons were performed using the Chi-square test. The correlation between two variables was analysed using Pearson's linear correlation analysis. Receiver operating characteristic (ROC) curves were generated to evaluate the diagnostic performance of ELA/Ang II and Ang (1-7)/Ang II ratios among the three groups. The area under the curve (AUC), sensitivity, specificity and optimal cut-off values were calculated, and AUCs were compared using DeLong's test. *P* < 0.05 was considered statistically significant.

## 3 Results

### 3.1 Baseline characteristics

As shown in Table 1, there were no statistical differences in age and gender composition among the three groups: healthy controls, AHF and Type I CRS. There was no difference in the aetiology of primary cardiac diseases between the AHF and CRS groups. Additionally, there were no statistical differences in the mean systolic blood pressure, mean diastolic blood pressure and mean heart rate among the control, AHF and CRS groups. There were no significant differences in total cholesterol and low-density lipoprotein cholesterol levels among the three groups. Compared to the healthy control group, the mean serum NT-proBNP levels were significantly higher in the AHF and CRS groups. The mean creatinine and blood urea nitrogen (BUN) levels in the CRS group were significantly higher than those in the control and AHF groups, while the estimated glomerular filtration rate (eGFR) level was significantly lower than that in the control and AHF groups. The mean LAD, LVEDD and LVESD were significantly higher in the AHF and CRS groups than in the control group, while the mean LVEF values were lower in both groups.

**Table 1:** The baseline characteristics of patients and controls.

Factors	Control (*N* = 31)	AHF (*N* = 31)	CRS (*N* = 38)	*P* value
Age	68.9 ± 9.6	69.1 ± 12.4	73.9 ± 11.2	0.106
Male(%)	22 (71.0%)	19 (61.3%)	25 (65.8%)	0.723
Female(%)	9 (29.0%)	12 (38.7%)	13 (34.2%)	0.723
Pathogeny				
Ischemic cardiomyopathy		9 (37.5%)	16 (48.5%)	0.409
Non-ischemic cardiomyopathy		15 (62.5%)	17 (51.5%)	0.409
SBP(mmHg)	126.6 ± 13.0	123.4 ± 20.4	129.8 ± 20.6	0.354
DBP(mmHg)	78.1 ± 10.9	76.8 ± 15.3	80.1 ± 12.9	0.587
HR(bpm)	72.0 (66.0,81.0)	80.0 (70.0,98.0)	77.0 (64.0,83.0)	0.381
NT-proBNP (pg/mL)	75.6 (32.6,148.5)	2,436 (1363,6547)*	4,260.5 (2707,14224)*	＜0.001
Scr(umol/L)	69.0 (60.0,75.0)	75.0 (60.0,86.0)	134.0 (104.5,174.3)*,**	＜0.001
BUN(mmol/L)	5.7 (4.4,6.3)	6.4 (5.5,7.3)	11.2 (8.3,16.4)*,**	＜0.001
UA(umol/L)	323.6 ± 85.3	422.2 ± 189.8*	472.2 ± 202.9*	0.002
eGFR(mL/min/1.73m2)	93.3 ± 9.9	88.1 ± 14.9	46.4 ± 18.5*,**	＜0.001
TC(mmol/L)	4.2 ± 1.0	4.1 ± 1.3	3.6 ± 1.1*	0.082
TG(mmol/L)	1.6 (1.1,2.5)	1.1 (0.8,1.4)	1.0 (0.8,1.5)*	0.024
HDL(mmol/L)	1.1 (1,0,1.4)	1.1 (0.9,1.3)	1.1 (0.9,1.3)	0.558
LDL(mmol/L)	2.0 (1.6,2.5)	2.2 (1.5,3.2)	1.8 (1.4,2.3)	0.249
Cardiac ultrasound				
LAD(mm)	34.2 ± 4.7	45.5 ± 9.0*	46.7 ± 10.0)*	＜0.001
RAD(mm)	34 (31.0,37.0)	43.0 (36.0,49.5)*	36.0 (30.0,44.0)	0.007
LVEDD(mm)	46.0 (44.0,48.0)	60.5 (51.3,68.0)*	56.5 (47.0,60.0)*	＜0.001
LVESD(mm)	31.0 (29.0,32.0)	49.0 (38.3,55.8)*	41.0 (32.0,48.0)*	＜0.001
RVD(mm)	32.5 ± 3.6	41.6 ± 10.6*	36.7 ± 6.6**	0.001
LVEF(%)	61.0 (60.0,63.0)	42 (37.3,50.5)*	51.0 (37.8,58.0)*	＜0.001

vs. Control *p < 0.05; vs. AHF **p < 0.05.

SBP, systolic blood pressure; DBP, diastolic blood pressure; HR, heart rate; NT-proBNP, N-terminal pro-brain natriuretic peptide; Scr, serum creatinine; BUN, blood urea nitrogen; UA, uric acid; TC, total cholesterol; TG, triglyceride; HDL, high density lipoprotein; LDL, low density lipoprotein; LAD, left atrial diameter; RAD, right atrial diameter; LVEDD, left ventricular end-diastolic diameter; LVESD, left ventricular end-systolic diameter; RVD, right ventricular diameter; LVEF, left ventricular ejection fraction.

### 3.2 Differences in the expression of ELA-APJ, ACE-Ang II and ACE2-Ang (1-7) among the three groups

Compared to the control group, the average concentration of Ang II in AHF patients slightly decreased. There was no significant statistical difference in the concentration of Ang II between patients with type I CRS and both AHF and control groups (AHF vs. Control, *P* = 0.0203, Figure 1A). Although there was a tendency for the concentration of ACE in AHF and type I CRS patients to rise in comparison to the control group, the difference was not statistically significant (Figure 1B). Compared to the control group, the average serum level of Ang (1-7) significantly increased in patients with type I CRS, and the average concentration of Ang (1-7) in the AHF group also increased compared to the control group (AHF vs. Control, *P* = 0.0258; CRS vs. Control, *P* = 0.0039, Figure 1C). The type I CRS group exhibited a significantly elevated average serum concentration of ACE2 compared to both the control group and AHF patients (CRS vs. Control, *P* < 0.0001; CRS vs. AHF, *P* = 0.0016, Figure 1D). Compared to the control group, the average serum concentration of ELA was significantly elevated in both AHF and type I CRS patients. Additionally, the average concentration of ELA in type I CRS patients decreased compared to AHF patients, but the difference was not statistically significant (AHF vs. Control, *P* < 0.0001; CRS vs. AHF, *P* = 0.0005, Figure 1E). There was no significant statistical difference in the concentration of APJ among the three groups (Figure 1F).

**Figure 1:**
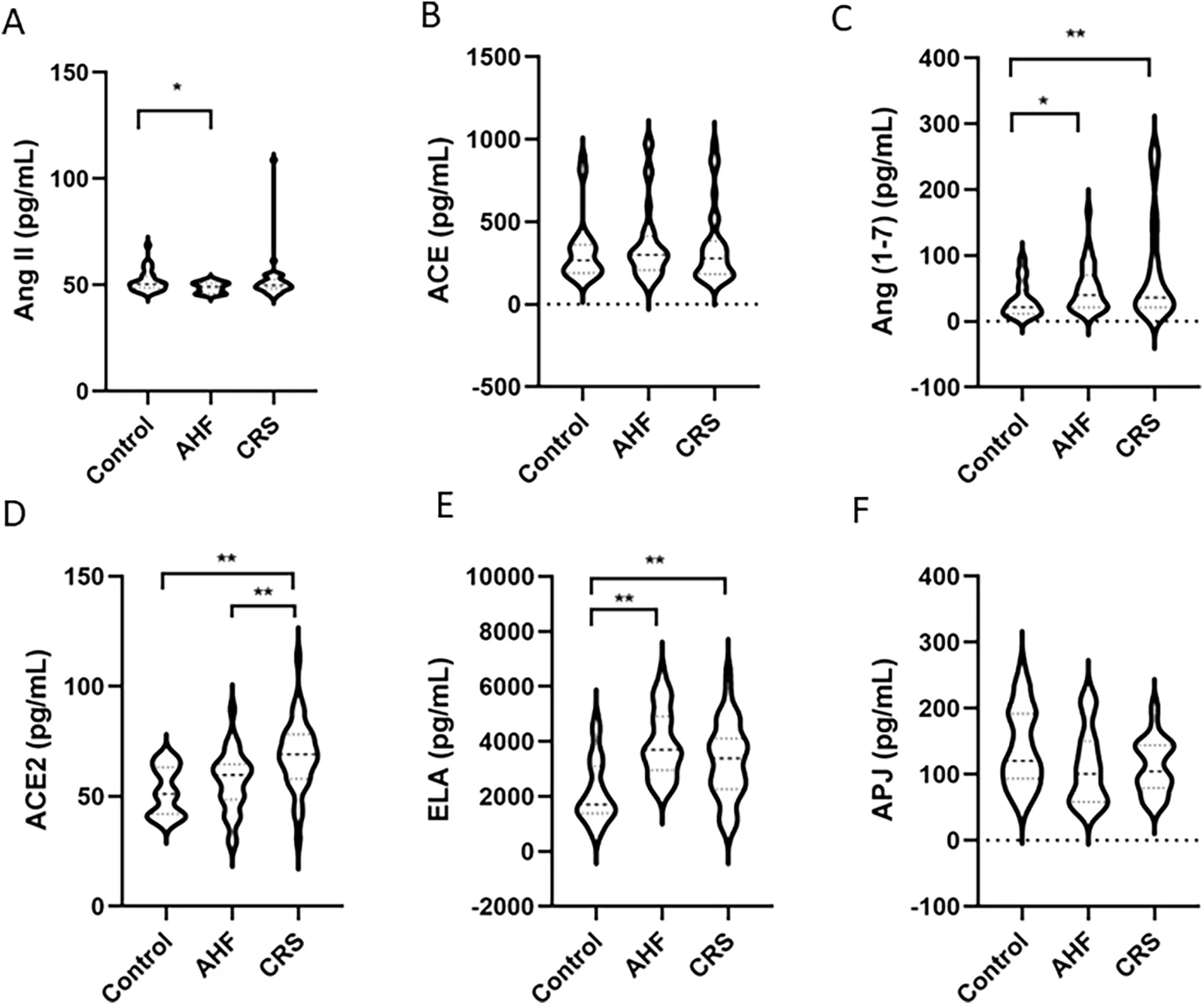
The endogenous expression of angiotensin II (Ang II) **(A)**, angiotensin-converting enzyme (ACE) **(B)**, angiotensin (Ang) (1-7) **(C)**, angiotensin-converting enzyme 2 (ACE2) **(D)**, elabela (ELA) **(E)** and apelin peptide jejunum (APJ) receptor **(F)** in health controls and patients. * *p* < 0.05; ** *p* < 0.01.

### 3.3 Differences in the expression of Ang (1-7)/Ang II and ELA/Ang II ratios among the three groups

Compared to the control group, the mean ratio of Ang (1-7)/Ang II increased in patients with AHF, and the ratio significantly increased in patients with type I CRS. No difference was observed in the ratio of Ang (1-7)/Ang II between the AHF and type I CRS groups (Figure 2A). Compared to the control group, the ratio of ELA/Ang II significantly increased in both the AHF and CRS groups. However, the ratio of ELA/Ang II was lower in the CRS group compared to the AHF group, with statistical significance (Figure 2B). Moreover, in the type I CRS group, the ELA/Ang II ratio negatively correlated with creatinine (r = −0.3559, *P* = 0.0283, Figure 2C) and positively correlated with eGFR (*r* = 0.5160, *P* = 0.0009, Figure 2D), while no significant correlation was observed between the ELA/Ang II ratio and NT-proBNP and LVEF. However, in the type I CRS group, no significant correlation was observed between the Ang (1-7)/Ang II ratio and cardiac and renal function indicators (Table 2).

**Table 2:** Correlation analysis between the ratios of Ang (1-7)/Ang II and ELA/Ang II and parameters related to cardiac and renal functions in type 1 CRS patients.

Factors	Ang(1-7)/Ang II ratio		ELA/Ang II ratio	
	r	p	r	p
Scr	-0.1169	0.4845	−0.3559	0.0283
BUN	−0.0505	0.7632	−0.2539	0.1240
eGFR	−0.1549	0.3530	0.5160	0.0009
NT-proBNP	0.1545	0.3545	0.0635	0.7050
LVEF	−0.1134	0.4980	0.2950	0.0722

Scr, serum creatinine; BUN, blood urea nitrogen; eGFR，estimated glomerular filtration rate; NT-proBNP, N-terminal pro-brain natriuretic peptide; LVEF, left ventricular ejection fraction. Ang (1−7), Angiotensin 1−7; Ang II, Angiotensin II; ELA, Elabela; CRS, cadiorenal syndrome.

**Figure 2:**
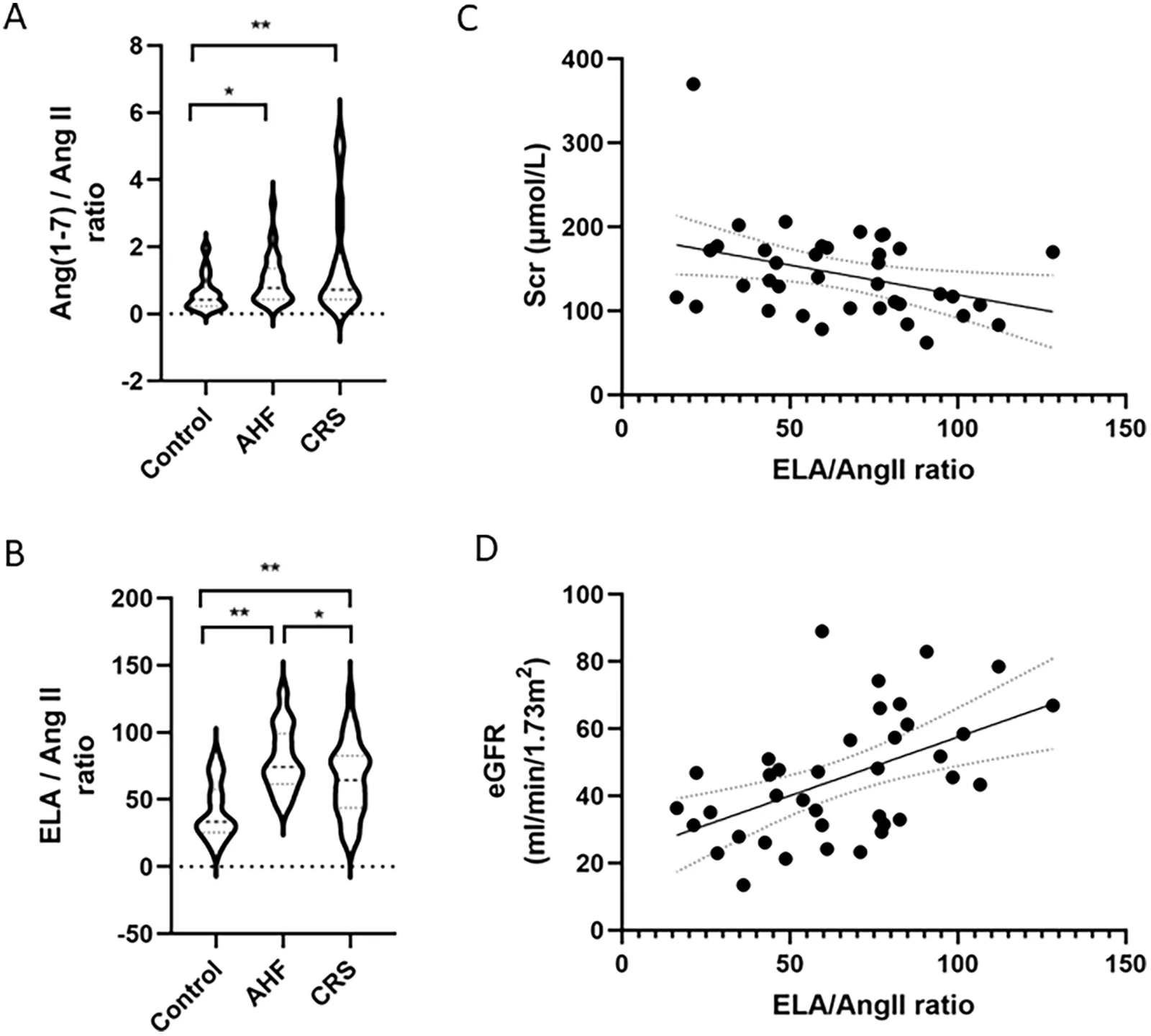
The mean ratios of Ang (1-7)/Ang II **(A)** and ELA/Ang II **(B)** among the three groups. The ELA/Ang II ratio negatively correlated with creatinine **(C)** and positively correlated with eGFR **(D)** in patients with type I cardiorenal syndrome (CRS). * *p* < 0.05; ** *p* < 0.01.

### 3.4 Diagnostic value of the Ang (1-7)/Ang II and ELA/Ang II ratios in AHF and type I CRS

The predictive ability of the Ang (1-7)/Ang II ratio for AHF was evaluated by the area under the ROC curve. The results showed that the AUC for the serum Ang (1-7)/Ang II ratio was 0.7128 [95% confidence interval (CI): 0.5842–0.8414, *P* = 0.004], with an optimal cut-off value of 0.415 for predicting AHF, thereby achieving a sensitivity of 87.10% and a specificity of 48.39% (Figure 3A). The AUC for the serum ELA/Ang II ratio was 0.8783 (95% CI: 0.7953–0.9612, *P* < 0.0001), with an optimal cut-off value of 54.73 for predicting AHF, thereby achieving a sensitivity of 90.32% and a specificity of 74.19% (Figure 3B).

**Figure 3:**
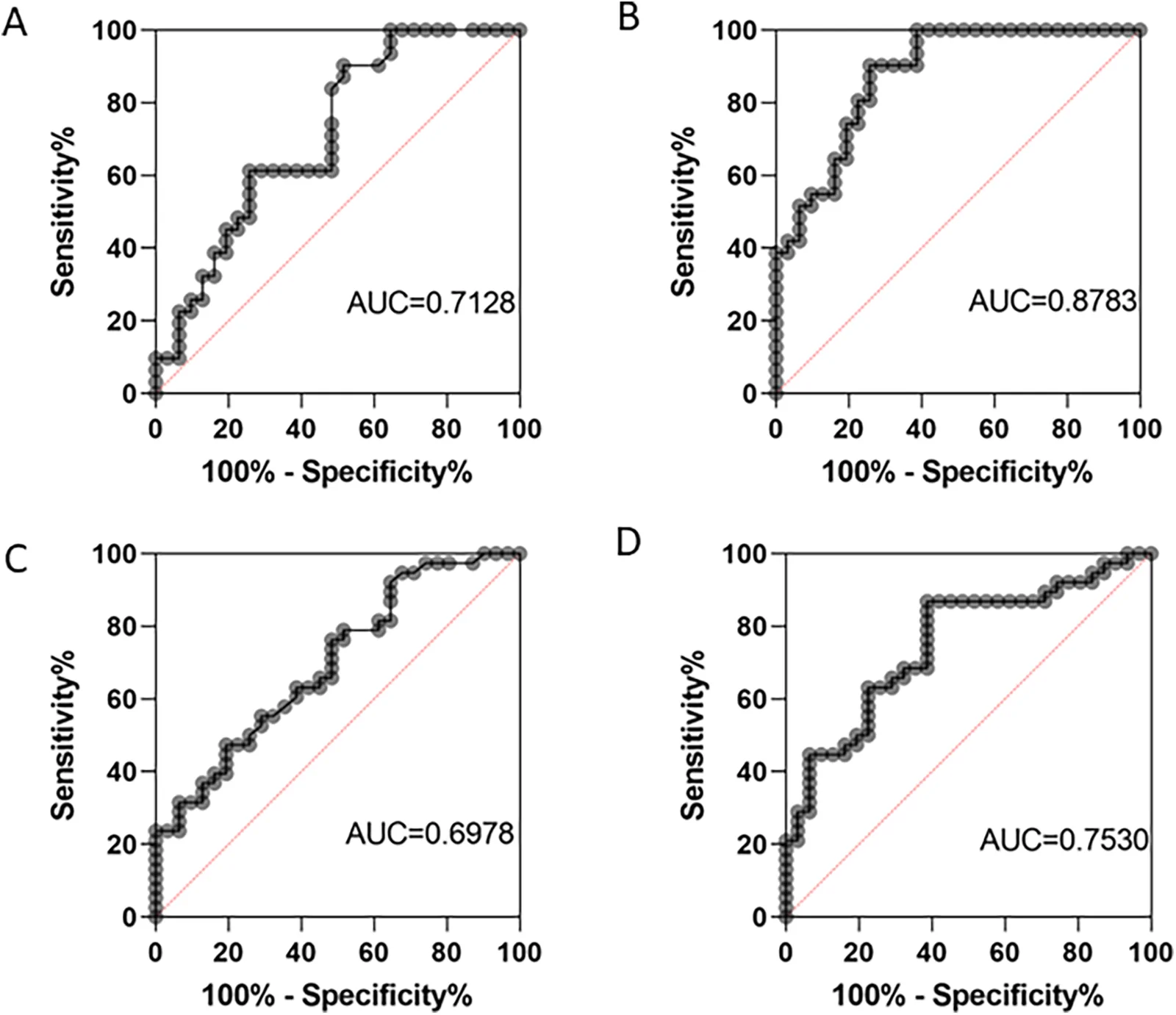
Receiver operating characteristic curves of ratios of Ang (1-7)/Ang II and ELA/Ang II for the diagnosis of acute heart failure (AHF) **(A,B)** and type I cardiorenal syndrome (CRS) **(C,D)**.

The predictive ability of the Ang (1-7)/Ang II and ELA/Ang II ratios for type I CRS was evaluated by the area under the ROC curve. The results showed that the AUC for the serum Ang (1-7)/Ang II ratio was 0.6978 (95% CI: 0.5752–0.8204, *P* = 0.0049), with the optimal cut-off value for predicting type I CRS being 0.43, at which the sensitivity was 76.32% and the specificity was 51.61% (Figure 3C). The AUC for the serum ELA/Ang II ratio was 0.7530 (95% CI: 0.6381–0.8678, *P* = 0.0003), with the optimal cut-off value for predicting type I CRS being 34.39, at which the sensitivity was 86.84% and the specificity was 61.29% (Figure 3D).

## 4 Discussion

The classical RAS system consists of ACE-AngII-AT1R. In this system, ACE converts Ang I into Ang II, which binds to AT1R to constrict blood vessels, promote the release of neurotransmitters from the sympathetic nervous system, stimulate aldosterone synthesis and release and act on the central nervous system to increase blood pressure. The non-classical RAS system consists of ACE2-Ang (1-7)-MasR. ACE2 converts Ang II into Ang (1-7), exerting a vasodilatory effect. The classical and non-classical RAS systems interact and counteract each other, and jointly participate in regulation of fluid balance and vascular homeostasis (21).

Herein, we found that compared to healthy controls, circulating Ang (1-7) concentration increased in patients with AHF, while Ang II serum levels slightly decreased; the Ang (1-7)/Ang II ratio increased. In type I CRS patients, Ang (1-7), ACE2 and the Ang (1-7)/Ang II ratio were significantly elevated compared to healthy controls, with ACE2 concentration also higher than in the AHF group. These results suggest that when AHF occurs or is complicated with acute renal insufficiency, the balance between the two main axes of the RAS is disrupted, leading to a compensatory increase in Ang (1-7). Cardiac remodelling is the fundamental process and pathophysiological basis of heart failure. The continued activation of Ang II-AT1R is one of the factors involved in the pathophysiological mechanism of cardiac remodelling. Ang II can promote cardiomyocyte hypertrophy, myocardial fibrosis, inflammatory response and oxidative stress (7–9).

In rat models, Ang II infusion significantly increases blood pressure, myocardial hypertrophy and myocardial interstitial fibrosis, while Ang (1-7) treatment can alleviate myocardial hypertrophy and interstitial fibrosis (22). Zhong J et al. found that under elevated Ang II conditions, ACE2 knockout mice exhibit exacerbated ventricular hypertrophy and myocardial fibrosis, leading to worsening of diastolic dysfunction. Recombinant human ACE2 (rhACE2) treatment reduces Ang II-mediated hypertension, ventricular hypertrophy and fibrosis, improving diastolic dysfunction. These beneficial effects of ACE2 are related to its ability to reduce Ang II or increase Ang (1-7) levels (23). *In vitro* experiments have shown that benzoylaconitine, an ACE2 agonist, can reduce Ang II-induced cardiomyocyte hypertrophy and fibrosis (24). The above studies indicate that targeting the ACE2-Ang (1-7) system can improve cardiac function and cardiac remodelling, making it a potential target for future heart failure treatment. Hisatake et al. found that serum ACE2 levels were elevated in AHF patients compared to healthy controls, with no significant differences in Ang II and Ang (1-7) levels (25). However, our research results showed that Ang II levels were slightly reduced in patients with AHF, while Ang (1-7) levels were significantly elevated. Moreover, ACE2 levels were significantly elevated in patients with AHF and renal insufficiency. The differences in research results may be attributed to variations in sample size, differences in the severity of cardiac and renal functions among enrolled patients and ethnic differences.

Previously, the expression differences of the Ang (1-7)/Ang II ratio in patients with different cardiac function classifications and their prognostic evaluation values have been explored. Basu et al. found that the Ang (1-7)/Ang II ratio in heart failure patients with New York Heart Association (NYHA) classifications of NYHA I/II was significantly higher than that in patients with NYHA III/IV, without any correlation to ejection fraction (EF) (26). Wang et al. enrolled 110 patients with heart failure and followed them for an average of 5.1 years. The results showed that an elevated Ang (1-7)/Ang II ratio was independently associated with the prognosis of heart failure patients (27). In the current study, we found that the Ang (1-7)/Ang II ratio was elevated in patients with AHF and type I CRS, while the value of Ang (1-7)/Ang II ratio in evaluating clinical prognosis needs further investigation.

Recently, the biological effects of the novel APJ ligand ELA in the cardiovascular system have been extensively studied. Our preliminary animal experiments suggest that ELA improves cardiac and renal function in mice with myocardial infarction and type I CRS through various mechanisms, such as anti-myocardial cell apoptosis, promoting angiogeesis and inhibiting inflammatory responses (28, 29). A previous clinical trial involving 69 patients with chronic congestive heart failure found a certain correlation between serum ELA levels and the sevenrity of cardiac function (30). Herein, we observed patients with AHF and type I CRS and found that compared to healthy controls, patients with AHF and type I CRS had significantly higher ELA levels and ELA/Ang II ratios; however, the ELA/Ang II ratio in patients with type I CRS was lower than that in the AHF group. Previous studies have shown that APJ is widely expressed in various human tissues and organs, while ELA is highly expressed in human kidney tissues. Furthermore, ELA mRNA expression was detected in glomerular epithelial cells and proximal tubular cells, suggesting that ELA may be mainly secreted by intrinsic kidney cells and act on various tissues and organs through the systemic circulation (31, 32).

Apelin and ELA, both endogenous ligands of APJ, exhibit similar physiological functions. Together with APJ, ELA/Apelin constitutes the apelinergic system, which plays crucial biological roles in maintaining fluid balance and regulating blood pressure in the body (33). Recent studies have shown that there is an antagonistic effect between the ELA-APJ and RAS systems. The ELA system can inhibit ACE expression, thereby limiting the production of Ang II. In contrast, Apelin can upregulate ACE2 expression, promoting the hydrolysis of Ang II to Ang (1-7) and subsequently reducing Ang II levels (18). These studies indicate that apelinergic system is interconnected and interacts with the ACE-Ang II and ACE2-Ang (1-7) systems, antagonising the overactivated RAS system. Basic research reveals that the binding of ELA/Apelin to APJ receptors activates signalling pathways such as phospholipase C, protein kinase C and ERK1/2, exerting a positive inotropic effect, enhancing myocardial contractility in failing hearts and increasing cardiac output (14, 34). These studies suggest that the ELA-APJ system represents a novel potential target for the future treatment of heart failure.

In the current study we found that ELA levels and the ELA/Ang II ratio were significantly higher in patients with AHF and type I CRS. Based on previous literature, we speculate that during acute cardiac dysfunction, the rise in ELA helps counteract the overactive RAS and enhances myocardial contractility in a failing heart. However, when AHF is accompanied by acute renal insufficiency, namely type I CRS, the ELA/Ang II ratio is significantly reduced compared to AHF patients without acute renal impairment. In a study on diabetic nephropathy, subjects were divided into three groups based on the level of albumin in 24-hour urine: normal albuminuria, microalbuminuria and macroalbuminuria. Serum ELA expression gradually decreased across these three groups. ELA significantly negatively correlated with serum BUN and positively correlated with eGFR, indicating that serum ELA levels gradually decrease with the severity of renal damage (35). Herein, we also found that the ELA/Ang II ratio in patients with type I CRS significantly positively correlated with eGFR. In patients with type I CRS, acute cardiac dysfunction may lead to decreased renal artery perfusion. This results in overactivation of the RAS and sympathetic nervous system, stimulation by inflammatory factors, and other contributors to acute renal ischaemia and hypoxia. Consequently, intrinsic renal cells secrete less ELA, reducing the endogenous ELA level (36).

Herein, we applied the ROC curve to observe the diagnostic value of Ang (1-7)/Ang II and ELA/Ang II for AHF and type I CRS. The results showed that the ratio of ELA/Ang II had more certain diagnostic value for AHF. The ratio of ELA/Ang II had predictive value for type I CRS. When the optimal cut-off value was 34.39, the sensitivity for diagnosing type I CRS was 86.84%. Considering the significant positive correlation between the ELA/Ang II ratio and eGFR in patients with type I CRS, it suggests that ELA/Ang II can be used as an additional means for diagnosing type I CRS.

The present study has several limitations. First, this is a cross-sectional observational study without external validation and prospective follow-up. Second, the results presented may be limited to serum levels and may not reflect the levels and activity of the Apela/APJ, ACE/Ang II and ACE2/Ang (1–7) systems in cardiovascular or other solid tissues. Third, the patients with heart failure and type I CRS in this study were not analysed by different NYHA grades and chronic kidney disease stages. Therefore, the practical application value of the ratios needs further confirmation through large-sample, prospective, randomised controlled studies.

## 5 Conclusions

The ELA/Ang II ratio can be used as an additional indicator for diagnosing AHF or type I CRS. Although the ELA/Ang II ratio is particularly well correlated with eGFR in patients with type I CRS, the value of the ratio in evaluating the degree of renal impairment in these patients remains to be further studied.

## Data Availability

The original contributions presented in the study are included in the article/Supplementary Material, further inquiries can be directed to the corresponding author.
